# Enhancing nursing and other healthcare professionals' knowledge of childhood sexual abuse through self‐assessment: A realist review

**DOI:** 10.1002/cesm.70019

**Published:** 2025-07-23

**Authors:** Olumide Adisa, Katie Tyrrell, Katherine Allen

**Affiliations:** ^1^ Violence and Society Centre City St George's University of London London United Kingdom; ^2^ Directorate of Learning and Teaching University of Suffolk Suffolk United Kingdom; ^3^ Institute of Social Justice and Crime University of Suffolk Suffolk United Kingdom

**Keywords:** child abuse, sexual, domestic violence, intimate partner violence, psychological trauma, self‐assessment

## Abstract

**Aim:**

To explore how child sexual abuse/exploitation (CSA/E) self‐assessment tools are being used to enhance healthcare professionals' knowledge and confidence.

**Background:**

Child sexual abuse/exploitation is common and associated with lifelong health impacts. In particular, nurses are well‐placed to facilitate disclosures by adult survivors of child sexual abuse/exploitation and promote timely access to support. However, research shows that many are reluctant to enquire about abuse and feel underprepared for disclosures. Self‐assessment provides a participatory method for evaluating competencies and identifying areas that need improvement.

**Evaluation:**

Researchers adopted a realist synthesis approach, searching relevant databases for healthcare professionals' self‐assessment tools/protocols relevant to adult survivors. In total, researchers reviewed 247 full‐text articles. Twenty‐five items met the criteria for data extraction, and to assess relevant contexts (C), mechanisms (M) and outcomes (O) were identified and mapped. Eight of these were included in the final synthesis based on papers that identified two key ‘families’ of abuse‐related self‐assessment interventions for healthcare contexts: PREMIS, a validated survey instrument to assess HCP knowledge, confidence and practice about domestic violence and abuse (DVA); Trauma‐informed practice/care (TIP/C) organisational self‐assessment protocols. Two revised programme theories were formulated: (1). Individual self‐assessment can promote organisational accountability; and (2). Organisational self‐assessment can increase the coherence and sustainability of changes in practice.

**Conclusions:**

There is a lack of self‐assessment tools/protocols designed to improve healthcare professionals' knowledge and confidence. Our review contributes to the evidence base on improving healthcare responses to CSA/E survivors, illustrating that self‐assessment tools or protocols designed to improve HCP responses to adult survivors of CSA/E remain underdeveloped and under‐studied. Refined programme theories developed during synthesis regarding DVA and TIP/C‐related tools or protocols suggest areas for CSA/E‐specific future research with stakeholders and service users.

## 1 INTRODUCTION

Child sexual abuse/exploitation (CSA/E) is a significant and costly public health issue in the United Kingdom [1, 2, 3]. The Centre of Expertise on Child Sexual Abuse estimates that approximately 500,000 children are sexually abused every year and that there is a widening gulf between reported cases and “hidden” abuse that has not been disclosed to or discovered by any official agency ([4]:13).

## 2 BACKGROUND

Nurses and other healthcare professionals are a trusted “first port of call” for adult survivors of CSA/E and should be well placed to facilitate earlier disclosures and access to specialist support [5, 6, 7]. While still an emerging body of research, studies that explore the acceptability of enquiry about child sexual abuse/exploitation and other forms of childhood adversity in primary care settings suggest that screening by clinicians is widely acceptable to patients [5, 8, 9]. However, healthcare professionals are hesitant to screen for child sexual abuse/exploitation and feel under‐prepared to deal with disclosures [10], despite a robust association with enduring, adverse health impacts and ‘revolving door’ health service use [11, 12, 13, 14, 15]. Meanwhile, child sexual abuse/exploitation survivors report a pattern of missed opportunities, where healthcare professionals failed to ask relevant questions and left them shouldering the “burden of disclosure” ([7]: 46).

This pattern of findings points to a need for targeted educational interventions to improve healthcare professionals' knowledge and confidence about child sexual abuse/exploitation. This is particularly relevant for nurses working in primary care and carrying out routine medical examinations and procedures, who will regularly encounter survivors during their work. Research with CSA/E survivors shows that these contact points offer a crucial opportunity for rapport‐building, facilitating disclosure and signposting to support (Schachter et al., 2009; [1, 7, 16]). However, medical contexts and examinations can also be triggering and potentially re‐traumatising for survivors (Schachter et al., 2009; [17]).

Self‐assessment potentially offers a pragmatic approach to highlighting knowledge deficits, and its participatory aspects may make it especially suitable for addressing sensitive subject matter, challenging attitudes or engrained elements of practice. To explore how self‐assessment tools are currently being used to increase health care professionals' knowledge and confidence about child sexual abuse/exploitation and under what circumstances and for whom such tools have proven effective, researchers conducted a realist review of the literature. This review represents a significant contribution to the literature on the use of self‐assessment tools, underlining the lack of targeted child sexual abuse/exploitation interventions currently available and pointing to fruitful areas for future research with stakeholders (including survivors).

### 2.1 Defining self‐assessment

Self‐assessment is “a personal evaluation of one's professional attributes and abilities against perceived norms” ([18]: 125).

To play to their strengths and remedy their knowledge deficits, competent professionals “need to know what they know with confidence, and similarly know what they don't know” (Ibid). In nursing, ongoing self‐assessment forms part of a broader culture of reflective practice and continuing professional development, which is crucial for “maintaining person‐centred, safe and effective evidence‐informed care” ([19]: 1).

For the review, researchers adopted a broad working definition of self‐assessment, which encompasses structured (individual and organisational) self‐evaluations of:

- Knowledge and skills.
- Confidence or self‐efficacy.
- Resources and capacities (including established policies and procedures, institutional environment, etc.).

### 2.2 Developing an initial programme theory

Preliminary scoping searches indicated that while there is an extensive body of research on health service responses to child sexual abuse/exploitation and a wealth of research on self‐assessment in medical education (see [18, 20, 21]), literature at the intersection of these two topics was sparse. Researchers, therefore, drew on a prior knowledge of UK healthcare contexts, relevant contextual insights from the literature on self‐assessment and the (separate) literature on child sexual abuse/exploitation, the expertise of our advisory group and middle‐range theories such as the normalisation process theory.

Researchers hypothesized that self‐assessment tools highlight healthcare professionals' knowledge deficits or deficiencies in practice in the proper organizational context. This leads to identifying areas for curricular standardization/improvement, individual learning needs, or areas of sub‐optimal practice as an organization. Possible contextual barriers identified included a culture of risk aversion and resistance to change and resource‐constrained health services. In contrast, facilitators included active leadership with an appetite for institutional innovation and improvement, a willingness to re‐direct staff time and resources or modify policies and procedures in response to self‐assessment ‘findings’, a culture of reflexivity and buy‐in by staff at all levels. This provided a theoretical framework for interpreting the literature and a working hypothesis to test against available evidence.

## 3 METHODS

Researchers conducted a realist review of the literature on the use of child sexual abuse/exploitation‐relevant self‐assessment tools in healthcare contexts. Following quality standards for realist reviews, the research questions were focused on iteratively. The protocol ([19],[22]) initially framed nine questions for investigation; after preliminary scoping and discussion of emerging findings, researchers refined these questions to focus on exploring how such tools are currently being used, the contexts (C) in which they are effective, and the mechanisms (M) by which they achieve observed outcomes (O). We set out to develop, test and refine a programme theory that would enable us to map what ‘good practice’ in implementing these tools could look like.

Our research protocol [22] and initial inclusion criteria and search strategy (see Inclusion Criteria and Search Strategy) outline our choice of approach and methodology in more detail. Following the development of the initial research protocol, which stipulates that EPPI‐Reviewer 4 will be used to support the synthesis, researchers opted to use the web‐based systematic review platform Covidence. The review process broadly adhered to Pawson's five stages for a realist review [23], including initial scoping and theory development, evidence searching, study selection and appraisal, data extraction, and evidence synthesis and analysis. This was an iterative and non‐linear process rather than a sequential set of steps.

### 3.1 Search strategy

In line with funding for the study, from February to March 2020, researchers conducted searches via 13 ProQuest databases and their institutional database for scholarly/peer‐reviewed articles and eBooks. The institutional database provides access to 189 e‐resources, including CINAHL Plus with Full Text, MEDLINE, PubMed and Science Direct. Relevant ProQuest databases searched include Applied Social Sciences Indexes and Abstracts (ASSIA), British Nursing Database, Health and Medical Collection, Healthcare Administration Database, Nursing and Allied Health Database, Psychology Database, Public Health Database and PTSD Pubs. Grey literature searches were conducted via the NICE health and social care evidence and WHO IRIS online repositories.

**Figure 1:**
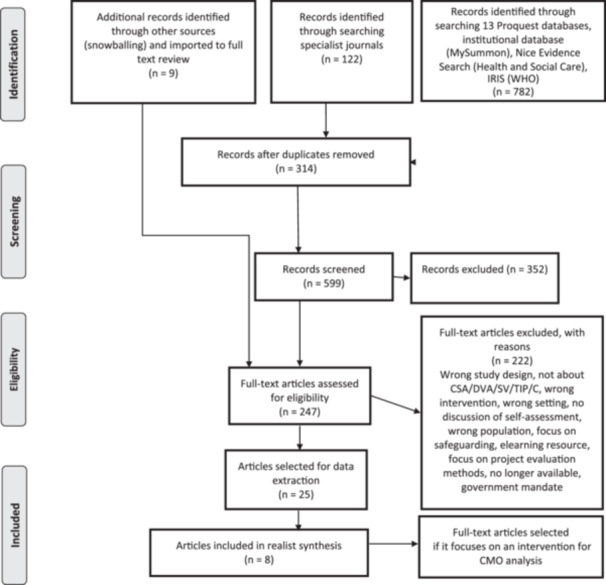
**A PRISMA flowchart of the realist review search and screening process**.

Search terms comprised combinations of the following key ‘intervention’ terms (“self‐assessment”, “self‐evaluation”, “audit tool”, “training”, “evaluation”) ‘setting’ terms (“health professional”, “health”, “healthcare”, “medicine”, “medic*”, “physician”, “clinical practice”, “medical education”) and ‘subject’ terms (“child abuse”, “child sexual abuse”, “child sexual exploitation”, “domestic violence”, “sexual violence”, “intimate partner violence”, “domestic abuse”, “trauma‐informed”). See the PRISMA flowchart below for further details regarding the screening and selection process. Figure 1

The research team imported 59 searches to Covidence, a web‐based systematic review platform,* comprising 599 unique search items. Three hundred fifty‐two items were excluded during title and abstract screening.

### 3.2 Iterative refinement of inclusion criteria and search strategy

**Table:** 

*Inclusion Criteria*
Literature discussing the use of child sexual abuse self‐assessment tools for health care professionals (including relevant non‐academic research such as grey literature and practitioner testimony). Literature discussing the use of domestic abuse self‐assessment tools for health care professionals (as above).
Literature discussing the use of sexual violence self‐assessment tools for health care professionals (as above).
*Exclusion Criteria*
Literature does not include discussion of child sexual abuse, domestic abuse, and/or sexual violence self‐assessment tools for health care professionals.
Abstract (or article/report summary, for non‐academic literature) not available in English.
Item published before 2000.

These initial inclusion and exclusion criteria were agreed upon in consultation with our multidisciplinary advisory group and chosen to afford a reasonable degree of ‘sensitivity’—erring on the side of inclusion rather than selectivity—while imposing pragmatic boundaries due to the dynamic nature of the review and size of the research team.

While the review was inspired by findings specifically about child sexual abuse/exploitation, in consultation with our advisory group, we decided from the outset that domestic abuse‐ and sexual violence‐related self‐assessment tools would also be relevant for inclusion. Both retrospective and longitudinal studies demonstrate a correlation between childhood experiences of sexual and/or physical abuse and subsequent victimisation by an intimate partner, with an influential association for child sexual abuse/exploitation: “Some have argued that sexual victimisation during childhood is among the strongest predictors of continued victimisation in adolescence and young adulthood” ([20],[24]: 413).

As the review progressed and gaps and emphases in the literature became more apparent, these criteria were further refined. We recognise that definitions of CSA and CSE may vary across the world; following consultation with advisory group members, it was agreed that CSE could represent a subset of child sexual abuse rather than a distinct phenomenon, and inclusion criteria were modified accordingly to incorporate relevant literature on both CSA/E. Subsequently, given researchers' focus on the long‐term impacts of child sexual abuse/exploitation and the costs associated with CSA/E, it was decided to exclude further papers that focus on child sexual abuse/exploitation survivors, which are, appropriately, predominantly oriented towards acute primary and secondary prevention and safeguarding, rather than managing chronic health impacts.

### 3.3 Study selection and quality appraisal

For the realist reviewer, the primary question for quality appraisal is, “Can this particular study (or fragment thereof) help, and is it of sufficient quality to help in respect of clarifying the particular explanatory challenge that the synthesis has reached?” ([25]: 135). That is, realist quality appraisals do not necessarily track or correspond to judgements of methodological rigour per se – which is why realist reviews are free to assimilate diverse sources, for example, grey literature.

To promote transparency and auditability, researchers documented their discussions, activities and decision‐making during the appraisal process using a jointly accessible logbook. Each of the 599 items imported to Covidence was reviewed and ‘voted’ on (on Covidence) by two reviewers: [first author] and [second author] reviewed most items, with assistance from [third author]. For each item where there were ambiguities or different assessments of relevance (e.g., if one reviewer voted ‘Maybe’ while the other voted ‘No’), items were reviewed in full and agreed upon jointly.

Following title and abstract screening, researchers reviewed 247 items in full, of which 25 were taken to be sufficiently relevant for ‘full’ data extraction. Eight of these were included in the final synthesis as they related to a specific intervention that a CMO analysis could be applied to.

### 3.4 Data extraction and synthesis

During both the full‐text review and data extraction stages, researchers recorded the title, authors, date, location, methods, sample population, tool or protocol being assessed, findings, and implications or recommendations for practice of each item, using a custom data extraction template (available on request). Each of these 25 items was subsequently assessed for theory‐building relevance, that is, detailed contextual and implementation data. All items that included an in‐depth discussion of implementing self‐assessment tools as an intervention were analysed for context‐mechanism‐outcome configurations (CMOcs).

Since the review was conducted in 2020 and following a helpful suggestion from one of the peer reviewers, we performed an additional hand search to identify any papers that may have utilised the two main ‘families’ of interventions included in the realist synthesis, namely PREMIS and/or Trauma‐informed practice/care (TIP/C) organisational self‐assessment protocols. To our knowledge, no papers have been published since 2021 that have used both self‐assessment tools, highlighting the importance of this paper in synthesising the existing evidence.

## 4 RESULTS

Following advanced data extraction, including appraisal of relevance for theory‐building/refinement and CMOc extraction, researchers found two distinct families of child sexual abuse/exploitation‐relevant self‐assessment tools in use in healthcare contexts: five studies used a domestic abuse‐related self‐assessment survey for use by individual health care professionals, while three articles employed organisational‐level self‐assessments to facilitate trauma‐informed practice/care. The domestic abuse‐related tool was implemented in a UK (*n* = 2) and US (*n* = 3) healthcare context, while the trauma‐informed practice/care protocol/workshops were used in US (*n* = 2) and Australian (*n* = 1) mental and behavioural health programmes and family services (Insert Data Extraction Table 1).

**Table 1:** Study Characteristics on self‐assessment tools use in healthcare settings.

Item reviewed	Self‐assessment tool used	Location	Methodology	Sample/population discussed	Findings	Implications/recommendations
Barnett Brown, V., Harris, M. & Fallot, R. (2013). Moving toward Trauma‐Informed Practice in Addiction Treatment: A Collaborative Model of Agency Assessment. *Journal of psychoactive drugs*, 45 (5): 386	Trauma‐informed self‐assessment and walk through protocol (Fallot & Harris)	US	Qualitative – theoretical article with case studies	Addiction programmes in the US	Between 30‐50% of individuals with substance use disorders have lifetime diagnoses of PTSD. Trauma is prevalent and causally linked to behavioural health issues. Yet involuntary, coercive and non‐gender responsive techniques such as restraint, seclusion and male nurses conducting night checks risk re‐traumatising patients	Self‐assessment protocols can aid healthcare services in identifying and correcting potentially retraumatising policies and practices at minimal or no cost
Beckett, P., Holmes, D., Phipps, M., Patton, D. & Molloy, L. (2017). Trauma‐Informed Care and Practice: Practice Improvement Strategies in an Inpatient Mental Health Ward. *Journal of Psychosocial Nursing*, 55 (10). pp. 34‐38	Trauma‐informed self‐assessment protocol with trauma workshops	Australia	Qualitative – case study of applying self‐assessment protocol in mental health ward	27 bed acute admissions ward, Melbourne hospital	Implementing trainings, self‐assessment and staff working groups resulted in significant changes over three years: 80% reduction in seclusions, introduction of women‐only section of ward, reduction in pharmacological sedation	Given the traumagenic nature of MH issues, all mental health services should deliver trauma‐informed care
^26^ Brady, M. (2018). UK Paramedics Confidence in Identifying Child Sexual Abuse: A Mixed‐Methods Investigation*. Journal of Child Sexual Abuse* 27 (4): pp. 439‐458	Online survey – Likert five‐point attitudinal scale to measure confidence in ability to recognise, child sexual abuse, and female genital mutilation	UK	Mixed methods – survey and focus groups	276 UK paramedics	Current training often brief, generic and competing for time with other service priorities. Paramedics expressed low‐self‐efficacy in relation to their ability to recognise child sexual abuse/exploitation and female genital mutilation. Perception that Child sexual abuse/exploitation is rare and only prevalent among certain populations	Need for further in‐depth training, with focus on recognising diverse non‐physical indicators that abuse may be occurring, as well as dispelling misapprehensions about prevalence and risk factors
^10^ Colthart, I., Bagnall, G., Evans, A., Allbutt, H., Haig, A., Illing, J. & McKinstry, B. (2008). The effectiveness of self‐assessment on the identification of learner needs, learner activity, and impact on clinical practice: BEME Guide no. 10*. Medical Teacher* 30 (2). pp. 124‐145	Systematic review of self‐assessment as a tool for identifying learner needs, and improving learning activity, clinical practice and patient outcomes	Global – included studies from US, UK, Canada, Australia, Sweden and elsewhere	Systematic review	32 papers met inclusion criteria, including methodological quality requirements	No high‐quality papers provided insight into whether self‐assessment results in a learning activity change, or an accurate perception of learning needsOnly two papers considered whether self‐assessments can improve clinical practice or patient outcomes. Neither presented compelling evidence that self‐assessments contribute to improvements in either areaFindings in relation to subsidiary research questions showed that learners are more accurate in assessing their peers than themselves; that demographic factors such as sex are associated with more or less accurate self‐assessments, and that the less competent are liable to over‐estimate their performance. Self‐assessment of practical skills was more in line with peer/expert assessments than self‐assessment of knowledge/cognitive skills	There is a need for more high‐quality research in this area
Connor, P., Nouer, S., Mackey, S., Banet, M. & Tipton, N. (2011). Dental Students and Intimate Partner Violence: Measuring Knowledge and Experience to Institute Curricular Change. *Journal of Dental Education*, 75 (8). pp. 1010‐1019	PREMIS self‐assessment tool	US	Quantitative – survey	318 dental, medicine, nursing and social work students at the University of Tennessee	70% of survey respondents reported having had no intimate partner violence training prior to dental schoolThe entire cohort reported low levels of preparedness and knowledge for supporting survivors of intimate partner violence relative to other respondents	There is a strong need for a standardised intimate partner violence training curriculum for dental professionals
^13^ Davis, D., Mazmanian, P., Fordis, M, Van Harrison, R., Thorpe, K. & Perrier, L. (2006). Accuracy of Physician Self‐assessment Compared with Observed Measures of Competence: A Systematic Review. JAMA. 296(9). pp. 1094‐1102. https://doi.org/10.1001/jama.296.9.1094	Systematic review of evidence on accuracy of individual self‐assessments relative to peer/'objective' external measures	Studies from US, UK, Canada, Australia and New Zealand	Systematic review	17 studies relevant for inclusion	Positive associations between self‐assessed expertise and observed/demonstrated competence in seven domains, including in ‘highly specialised’ areas such as the identification of signs of child sexual abuse (a pre‐2000 study). They sub‐defined self‐assessments as either predictive, concurrent or summative	Develop more detailed learning and practice objectives to guide professionalsIncorporate ‘objective measurements or benchmarks of performance’, so that professionals are more attuned to what is expected of them and are better equipped to self‐assess performance and learning needsConsider incorporating ‘multisource feedback’ (360° evaluations) when assessing communication or interpersonal skillsDevelop external measures and guides to facilitate more accurate/useful self‐assessmentsA greater role for specialist societies, who could provide up to date, evidence‐based learning objectives
DeCorby‐Watson, K., Mensah, G., Bergeron, K., Abdi, S., Rempel, B. & Manson, H. (2018). Effectiveness of capacity building interventions relevant to public health practice: a systematic review. BMC public health 18 (1). pp. 684‐15	Systematic review of capacity building interventions	Global – English language literature published 2005 onwards	Systematic review	14 studies examining six intervention types:1) Internet‐based instruction2) Training and workshops3) Technical assistance4) Education using self‐directed learning5) Communities of practice6) Multi‐strategy interventions	Reviewers assessed outcomes related to learner knowledge, self‐efficacy, perceived support, changes in policies or practice, skills, or support environmentsInternet‐based instruction was found to be more effective than no intervention, but less effective than other interventions studiedSelf‐directed learning increased knowledge but not skillsCommunities of practice and traditional educational strategies produced the most significant improvements across impact domains	There is a need to strengthen the evaluation of capacity building interventions, particularly organisational and systems level interventions
^15^ Fetters, M. Motohara, S., Ivey, L., Narumoto, K., Sano, K., Terada, M., Tsuda, T. & Inoue, M. (2017). Utility of self‐competency ratings during residency training in family medicine education‐emerging countries: findings from Japan. *Asia Pacific Family Medicine*, 16 (1). https://doi.org/10.1186/s12930-016-0031-1	142 item online survey measuring self‐perceived competency in different subject areas within family medicine/general practice	Japan	Quantitative – cross‐sectional longitudinal study over a four‐year period	20 medical residents (11 women, nine men)	Scores improved annually from baseline to graduation, with the composite score across subject areas increasing from 31% to 65%. All subcategories showed improvement, with the greatest increase in women's health care, screening and geriatrics	Self‐assessment represents a feasible method of monitoring resident progress and inform programme development
^28^ Fraser, J., Griffin,., Barto, B. Lo, C., Wenz‐Gross, M., Spinazzola, J., Bodian, R., Nisenbaum, J. & Bartlett, J. (2014). Implementation of a workforce initiative to build trauma‐informed child welfare practice and services: Findings from the Massachusetts Child Trauma Project. *Children and Youth Services Review* 44. pp. 233‐242	Trauma‐informed self‐assessment protocol, with dissemination of trauma training and trauma‐informed leadership teams	US	Qualitative	192 clinicians and 1096 child welfare workers from 20 MH and social care agencies across Massachusetts	Following dissemination of trauma training and formation of Trauma Informed Leadership Teams, 298 children were enrolled in evidence‐based treatments. This fell short of enrolment goals	Issues such as time and budgetary constraints, high staff turnover due to burnout/vicarious trauma affect implementation and must be accounted for in planning and pilot stages
^27^ Horwood, J., Morden, A., Bailey, J., Pathak, N. & Feder, G. (2018). Assessing for domestic violence in sexual health environments: a qualitative study. *Sexually transmitted infections* 94 (2). pp. 88‐92	Evaluation of a pilot training intervention to promote domestic abuse screening – Identification and Referral to Improve Safety (IRIS)	UK	Qualitative – semi‐structured interviews	17 sexual health clinic staff and domestic abuse advocate workers	Most patients responded well to screening, particularly women. The addition of an automatic prompt to electronic patient records was seen as positive and impactfulStaff struggled with time constraints, the added administrative burden of evidencing/logging enquiry and other priorities (e.g. asking about smoking or alcohol use). There was a lack of available services to refer male victim‐survivors to	Interviewees emphasised the need for ongoing feedback and refresher training sessionsContextual barriers that emerged during interviews show that leadership buy in and support from NHS Trusts and commissioning groups are crucial to ensure sustainability and staff morale. Additional work associated with screening should be recognised and reimbursed, with time allowances if necessary
Jones, K. M. (2016). Obstetrician/gynaecologists' readiness to manage intimate partner violence. PhD thesis in Clinical Psychology. American University Washington DC	PREMIS self‐assessment tool	US	Quantitative	194 members of the American College of Obstetricians and Gynaecologists, 981 patients	*Only 20.2% of ob/gyn survey respondents reported routinely screening all patients for intimate partner violence* Non‐White patients were significantly more likely to be screened than White patients* First‐time patients were significantly more likely to be screened than returning patients	Research suggests that patients respond favourably or neutrally to routine enquiry, but physicians remain reluctant to screen. It is therefore imperative to address physician reported barriers to enquiry, such as discomfort about raising the subject of intimate partner violenceFuture training and educational programmes could emphasise patient satisfaction with routine intimate partner violence screening
Moskovic, C.S., Guiton, G., Chirra, A., Núñez, A., Bigby, J., Stahl, C., Robertson, C., Thul, E., Miller, E., Sims, A., Sachs, C., & Pregler, J. (2008). Impact of Participation in a Community‐Based Intimate Partner Violence Prevention Program on Medical Students: A Multi‐Centre Study. *Journal of General Internal Medicine*, 23. pp. 1043‐1047	Pre‐post survey to test actual knowledge, attitudes and confidence	US	Quantitative/randomised controlled trial. Students randomly assigned to didactic training with or without participation in community‐based programme	117 students attending four medical schools	Both conditions demonstrated significant increase in knowledge and a greater increase in confidence for participants who completed didactic training, whether assisted with community‐based intimate partner violence programme or not	Survey instrument suggests self‐reported confidence not only contingent on theoretical knowledge but on practical experience
Murray, H. (2017). Evaluation of a Trauma‐Focused CBT Training Programme for IAPT services. *Behavioural and Cognitive Psychotherapy* 45 (5). pp. 467‐482	Pre‐post training self‐rated PTSD competencies on an adapted/bespoke assessment tool	UK	Mixed methods – actual knowledge, self‐reported competencies, training feedback, supervisor feedback, patient outcomes	20 therapists from 10 IAPT services	The training programme was successful in improving trauma‐focused CBT knowledge, skills and outcomes. Feedback from participants indicated that they found the training highly acceptable	High staff turnover and institutional barriers within participating services suggest that sustainability is contingent on trained staff remaining with services and successfully disseminating training
^14^ Pierides, K., Duggan, P., Chur‐Hansen, A. and Gilson, A. (2013). Medical student self‐reported confidence in obstetrics and gynaecology: development of a core clinical competencies document. *BMC Medical Education*, 13	Core competencies list – administered as part of an 81‐item survey	Australia	Mixed methods – a candidate list of core competencies was reviewed at two focus groups, then administered as online survey	172 medical students	Confidence in history‐taking skills was low in relation to patients presenting with signs of sexual violence or abuse (less than 46%)	Open ended responses suggested class sizes and a lack of clinical experience adversely impacted confidence. More research needed in these areas
Ramsay, J., Rutterford, C., Gregory, A., Dunne, D., Eldridge, S., Sharp, D. and Feder, G. (2012). Domestic violence: knowledge, attitudes, and clinical practice of selected UK primary healthcare clinicians. *British Journal of General Practice*. https://doi.org/10.3399/bjgp12X654623	PREMIS self‐assessment tool	UK	Quantitative – prospective observational cohort study	272 clinicians from 48 general practices in Hackney and Bristol (59% response rate)	Findings showed that most participants had only minimal previous domestic abuse training and basic knowledge. Low frequency of enquiry/screening in response patients who present with possible indicators that they are experiencing abuse	Both GPs and practice nurses require more training on domestic abuse, including available support services locally
Ritchie, M, Nelson, K. Wills, R, & Jones, L. (2014). Development of an Audit Tool to Evaluate the Documentation of Partner Abuse Assessments within a Provincial Emergency Department: An Exploratory Study. *Journal of Family Violence* 29 (2): pp. 215‐221	Clinical audit tool	New Zealand	Mixed methods – five stage development process, including systematic review, selecting review criteria, piloting and testing for inter‐rater reliability	Documentation of women aged 16+ attending hospital emergency department for intimate partner violence		Quality assessment and documentation are vital to support effective intervention with patients who have experienced intimate partner violence
Ritchie, M., Nelson, K., Wills, R. & Jones, L. (2013). Does Training and Documentation Improve Emergency Department Assessments of Domestic Violence Victims? *Journal of Family Violence*, 28 (5): pp. 471‐477	Clinical audit tool	New Zealand	Quantitative – 80 randomly selected clinical records from a nine‐year period scored using Family Violence Identification Form (FVIF) and scores entered into the Statistical Package of Social Science Research (v18)	80 clinical records of women aged 16+ attending emergency department for intimate partner violence	Trends throughout the stages of programme development showed that training alone led to no improvement in assessment or documentation – training plus the introduction of the FVIF yielded significant improvement	In order to successfully implement change, leadership need to understand the barriers and facilitators to improving practice; for example, why staff members may feel hesitant to ask about intimate partner violence
^22^ Short, L., Alpert, E., Harri, J. & Surprenant, Z. (2006). A tool for measuring physician readiness to manage intimate partner violence*. American Journal of Preventive Medicine* 30 (2). pp. 173‐180	PREMIS self‐assessment tool	US	Quantitative – psychometric survey instrument development, testing and refinement	166 practicing physicians – subscribers to a continuing medical education website	The final 67 item survey tool demonstrated good internal consistency reliability, with Cronbach's alpha greater than or equal to 0.65 for 10 final scales. The developed scales were closely correlated with theoretical constructs and predictive of self‐reported behaviours.	The tool could contribute to effective healthcare responses to intimate partner violence victim‐survivors, by assessing HCP's knowledge and preparedness and identifying areas for improvement. Future studies should investigate the relationship between self‐reported KABB (knowledge, attitudes, beliefs and behaviours) physician behaviours, and patient outcomes
Sohal, A., Pathak, N., Blake, S., Apea, V., Berry, J., Bailey, J., Griffiths, C. and Feder, G. (2018). Improving the healthcare response to domestic violence and abuse in sexual health clinics: feasibility study of a training, support and referral intervention. *Sexually transmitted infections*, 94 (2): p. 83	Pre‐post training self‐assessment surveys (for Site 2 only, as low response rate at Site 1)	UK	Adaptive mixed methods pilot study – intervention comprised multidisciplinary training sessions electronic prompts,	Two women's walk in sexual health clinics	Self‐rated knowledge about domestic abuse health consequences, enquiry, response and advocacy referrals rose by 40%. All feasibility outcomes met	Further evaluation required to confirm intervention effectiveness prior to scaling up nationally
Songer, T., Stephens‐Stidham, S., Peek‐Asa, C., Bou‐Saada, I., Hunter, W., Lindemer, K. & Runyan, C. (2009). Prevention and Preparedness: Core Competencies for Injury and Violence Prevention. *American Journal of Public Health* 99 (4): pp. 600‐606.	Core competencies framework	US	Qualitative – consulted expert advisory panel and public to arrive at consensus on essential and desirable competencies for public health professional working in violence and injury prevention	52‐person expert panel, public comment from 32 relevant agencies	Consensus around nine key competencies:* Define and explain injury and violence as a social and health problem, including conceptual models and risk factors* Access, use, interpret and prevent violence and injury data* Design and implement prevention activities* Evaluate prevention activities* Build and manage a prevention programme* Stimulate change through policy, advocacy, enforcement and education* Maintain and develop competencies as a professional* Demonstrate competence in a specific violence or injury topic	Core competencies framework is designed to provide public health professionals working in violence or injury prevention with a standard set of skills for practice, to guide professional development and learning
Trevillion, K., Agnew‐Davies, R. and Howard, L. M. (2011). Domestic violence: responding to the needs of patients. *Nursing Standard* 25 (26): pp. 48‐56; quiz 58, 60	Self‐assessment questionnaire	UK	NA – article offering overview of domestic abuse facts and questionnaire	Guidance for nurses	NA	Promotes testing subject knowledge as a means of consolidating and directing learning
Williamson, E., Jones, S., Ferrari, G., Debbonaire, T., Feder, G. and Hester, M. (2015). Health professionals responding to men for safety (HERMES): feasibility of a general practice training intervention to improve the response to male patients who have experienced or perpetrated domestic violence and abuse. *Primary Health Care Research Development*. 16 (3): pp. 281‐288	PREMIS self‐assessment tool	UK	Mixed methods – pre‐post completion of PREMIS, disclosures documented in clinical records, semi‐structured telephone interviews	25 survey participants, 7 interviewees. All physicians from 4 general practices in Bristol	Post‐training surveys showed an increase in self‐reported ability to respond to disclosures and statistically significant improvements in perceived competence in responding to male patients	Further research needed to better understand men's help‐seeking behaviours. Clinician feedback shows a need to consolidate domestic abuse training to accommodate time constraints
World Health Organisation (2019). *Caring for women subjected to violence: a WHO curriculum for training health‐care providers*. Available at: https://www.who.int/reproductivehealth/publications/caring-for-women-subject-to-violence/en/	Training curriculum, including pre‐ and post‐training self‐assessment tools	Global, with a particular focus on low‐ and middle‐income countries	NA – 13 training sessions to deliver over two and a half days	NA – the training curriculum is primarily designed for primary healthcare providers	NA	The four objectives of the training are:1. Demonstrate general knowledge of violence against women as a public health problem2. Demonstrate behaviours and understand values contributing to safe and supportive services for survivors3. Demonstrate clinical skills appropriate to one's profession and specialty to respond to violence against women4. Demonstrate knowledge of how to access resources and support for patients and for oneself (3)
Royal College of Nursing (2017). National Curriculum and Competency Framework Emergency Nursing (Level 1)	Core competency framework	UK	NA	NA	NA	Recommendation to set realistic development goals at one‐to‐one meetings and revisit and review regularly. Meet with mentor at three‐month intervals during year 1, and then six‐month intervals during year 2 to review progress in core competencies
Royal College of Nursing (2017). National Curriculum and Competency Framework Emergency Nursing (Level 2)	Core competency framework	UK	NA	NA	NA	Recommendation to set realistic development goals at one‐to‐one meetings and revisit and review regularly. Meet with mentor at three‐month intervals during year 1, and then six‐month intervals during year 2 to review progress in core competencies

From the developer/implementers' stated programme theories, salient contextual information gleaned from all full‐text reviewed articles and middle‐range theories (Normalisation Process Theory), researchers isolated three central functions or uses of self‐assessment in healthcare contexts:

- ‘Primary’ self‐assessment, used to highlight areas where knowledge or confidence is lacking, or where health care professionals hold inaccurate or harmful beliefs and attitudes (Type 1).
- Organisational self‐assessment protocols used to identify areas for institutional transformation (Type 2).
- ‘Secondary’ or indicative self‐assessment/pre‐post testing used to measure the efficacy of an educational intervention (Type 3).

### 4.1 Highlighting individual knowledge and confidence deficits to create collective accountability

We hypothesised that self‐assessment tools affect educational and practice improvements via a highlighting mechanism – picking out areas where current knowledge and practice is lacking. As Table 2 indicates, identified CMOcs for the uses of PREMIS both support and enrich this model, contributing a more nuanced or dimensional understanding of how the proximal outcome (evidencing gaps or changes in knowledge, confidence and practice) can, in the right context, become a mechanism triggering meaningful organisational changes. Notably, none of the reviewed studies discussed PREMIS in the context of self‐directed learning by individuals (one of its suggested functions by developers [26], so this does not feature in our revised programme theory.

**Table 2:** CMOcs^a^ for PREMIS.^b^

Reference	Intervention and CMOc	Illustrative quote(s) with page number
Short et al. [26]	67 practicing primary care physicians in Phoenix and Kansas City (C) completed the revised PREMIS survey (following initial development and testing with 166 practicing physicians recruited via a CME website) (I). The results demonstrated variations in domestic abuse training between experienced physicians, as well as significant inter‐group differences in Perceived Preparedness and on one of the Opinion scales (Legal Issues) (M), highlighting areas where further and/or more effective domestic abuse training is needed (O).	“This instrument has the potential to be useful in a number of different ways: (1) as a pretest and needs assessment to measure physician knowledge, attitudes, beliefs, behaviors, and skills that may need to be addressed during training or other on‐site intervention; (2) as a training adjunct to orient physicians to the topic and expose them to the complexity of intimate partner violence^c^ issues; (3) as a post‐test to determine changes in physician KABB^d^ over time or as the result of training; and (4) as a comparative instrument to assess differences in KABB between physicians who have received training and those who have not” (p. 7).
Connor et al. (2011)	61 final‐year dental students at Tennessee Health Science Centre (C) completed a modified version of PREMIS (adapted for student populations) (I). The survey results highlighted considerable variations in training history (36% of participants reportedly received no domestic abuse training during their dental education) and deficiencies in the sample group's actual knowledge of domestic abuse (M1), pointing to areas for curricular standardisation and improvement (M2). Authors used these findings to inform an overhaul of the family violence curriculum (O)	Authors "found that a sizeable number (ranging from half to nearly two‐thirds) of our dental students who were preparing to enter the profession as practicing dentists are still receiving no education about the highly prevalent health problem of intimate partner violence. Although the trend in higher education continuesto support providing students with more and better training in intimate partner violence, our findings reflect research which affirms that there remains a pronounced deficit in intimate partner violence education across disciplines particularly in the field of dentistry […] At the institutional level, we also plan to incorporate student survey responses as part of a revision of the family violence curriculum" (p. 1017)
Ramsay et al. (2012)	PREMIS surveys were sent to two UK general practice surgeries: Bristol and Hackney (I). Across the two sites, there were 272 responses (64% response rate from Bristol and 50% response rate from Hackney) (C). The survey responses demonstrated a lack of perceived preparedness and actual knowledge in relation to domestic abuse, as well as a reluctance to directly enquire about abuse (M1). This demonstrated lack of knowledge provides evidence of the need for standardised, comprehensive training (M2). The authors advocate for improvement in this area, generating external pressure for improved domestic abuse training for general practice staff (O).	“This study highlights the persistent poor preparation of general practices for responding to the needs of women experiencing domestic violence. There is an urgent need for more comprehensive training at undergraduate and postgraduate levels and explicit referral pathways to specialist domestic violence services for women disclosing abuse.” (p. 654)
Williamson et al. (2015)	A modified version of PREMIS, (edited down to the sections on clinical practice and behaviours) was disseminated as a pre‐post intervention measure (I). 14 practitioners who attended training on supporting male patients experiencing or perpetrating domestic abuse completed the survey twice ‐ once before training and again at 6 months after the training. Participants were drawn from four general practice surgeries in Bristol (C). The survey functioned as a partial test/demonstration of the effectiveness of the training (in addition to external measures such as increased identification of domestic abuse) by tracking changes in perceived preparedness and competence (M1), providing researchers with supportive evidence that the training offers better outcomes than the ‘standard’ (M2). This adds to the evidence base supporting wider, evidence‐based domestic abuse training (O).	When administered 6 months post‐training, the modified PREMIS survey demonstrated “an improvement in perceived ability to manage situations where patients either disclosed, or the clinicians suspected, exposure to domestic abuse. There were statistically significant improvements in perceived competence in responding to heterosexual, gay, or bisexual male patients” (p. 284)
Jones (2016)	400 members of the American College of Obstetricians and Gynaecologists (C) were provided with two domestic abuse‐related surveys: PREMIS (for the physician's own completion) and 25 copies of the Patient Safety and Satisfaction Survey for completion by patients directly following an appointment (I). Survey responses supported the researcher's hypothesis that obstetricians and gynaecologists rarely screen for domestic abuse despite clear recommendations by professional bodies (M1), illustrating the need to educate physicians in the benefits of screening (M2). These findings add to the evidence base supporting wider, evidence based domestic abuse training (O).	“Our results indicate that screening rates have not improved in over 15 years despite public health and medical recommendations and empirical evidence demonstrating that screening is an effective way to identify intimate partner violence and intervene to support survivors of abuse […] Considering that routine intimate partner violence screening rates are still low among physicians, but satisfaction among patients who are screened is high (98% satisfied) […] it seems imperative to address physician reported barriers to intimate partner violence screening, such as discomfort in asking about intimate partner violence [… and] emphasize patient satisfaction with routine intimate partner violence screening” (pp. 46‐47)

^a^ Context‐mechanism‐outcome configuration.

^b^ Physician readiness to manage intimate partner violence survey.

^c^ Intimate partner violence.

^d^ Knowledge, attitudes, beliefs and behaviours.

By using PREMIS to diagnose individual learners' knowledge and/or perceived competence deficits, (I) implementers can use collated findings to identify and document areas where curricular improvement or standardisation is needed (M1) and create an evidence trail of the need for curricular change (M2) which, in a receptive organisational context (C), incentivises changes to the curriculum (O). M2, the implicit intervening mechanism, provides the missing step between pinpointing areas of ignorance and actually taking action to address these. By providing tangible evidence of deficiencies in current practice, PREMIS can act as an accountability mechanism by generating (internal or external) pressure, spotlighting areas where change is needed and providing the impetus to act. Conversely, by demonstrating collective gains in knowledge and confidence following training (M1), PREMIS supports the efficacy of the educational intervention being trialled (M2), which, in the right context (C), incentivises wider adoption of evidence‐based training strategies and phasing out of ineffective educational practices (O).

### 4.2 Organisational self‐assessment can increase ‘coherence’

Our review yielded fewer relevant articles on trauma‐informed practice/care organisational self‐assessments, and, in contrast to our review's focus on primary care and generalist services, the implementation contexts featured in these studies were settings more traditionally associated with trauma: child welfare and adult behavioural and mental health services (see Table 3). It is, therefore, possible that some identified causal mechanisms and barriers will not be directly transferable. However, there were significant similarities between contextual pressures observed in the study sites and those operating in primary healthcare settings, for example, time and resource constraints, service demands, staff turnover and burnout.

**Table 3:** CMOcs^a^ for TIP/C^b^ organisational self‐assessments.

Reference	Intervention and CMOc	Illustrative quote(s) with page number
Barnett Brown et al. (2013)	Research and clinical evidence show that the majority of patients in substance use programmes have histories of trauma and abuse, which can lead to negative treatment outcomes/poor retention (C). Fallot & Harris' self‐assessment and walkthrough protocol (2011) (I) enables service providers to assess how trauma‐informed their practice is at baseline, map where progress is needed and evaluate change following training (M1), helping service providers to understand how they can improve their programme for traumatised service users (M2). This understanding of current practice failings and their therapeutic significance motivates evidence‐based changes (O).	“Each of these [assessment] elements are utilized as a pre‐post measure in the assessment; that is, what does the agency look like at baseline and what does it look like after training and consultation has taken place […] The walk‐through is a mutual data‐gathering strategy that does not feel judgmental” (p. 388).
Beckett et al. (2017)	The Clinical Nurse Consultant and Senior Clinical Psychologist at a 27‐bed acute mental health ward in Metropolitan Sydney, Australia (C) facilitated a series of trauma‐informed workshops to embed the core values identified by Fallot & Harris (2011): choice, collaboration, trustworthiness, safety, and empowerment (I). Through these workshops, staff collaboratively identified six key practice areas that were insufficiently trauma‐informed (M). Working groups were mobilised to address these, effecting positive changes in practice for traumatised patients(O).	“The integration of strengths‐based philosophies and practice was reflected in a reduction in the use of clinical jargon and pejorative descriptions of consumers (e.g., chronic schizophrenic) and efforts to focus on consumer strengths and resources during clinical discussions and handover. Greater awareness of childhood and adult adversity encouraged greater understanding, compassion, and respect for consumers” (p. 36).
Fraser et al. [27]	The Massachusetts Department for Children and Families launched a state‐wide initiative to build capacity for trauma‐informed practice/care and trauma‐specific services, including the formation of trauma‐informed leadership teams (C). These teams performed an initial self‐assessment to identify issues with current practice and provide metrics for capturing change (M), which led to improvements in practice (O).	“By the end of the initial implementation year (September 2013), the majority of the teams in the Northern and Western regions had completed their self‐assessments and were implementing trauma‐informed innovations to address issues identified through the assessment process” (p. 238)

^a^ Context‐mechanism‐outcome configuration.

^b^ Trauma‐informed practice/care.

Normalisation process theory is a middle‐range theory sometimes used during intervention development, helping designers to anticipate and avoid “translational gaps” and enhance the potential for normalisation, or embedding within routine practice – for example, understanding which aspects of a complex intervention may have low coherence for implementors and working to make these aspects more meaningful and salient for them ([24][28]: 2). By offering a more collaborative approach to identifying knowledge gaps and shortcomings in current practice, we hypothesised that self‐assessment may promote positive behavioural change by increasing coherence and supporting sense‐making.

Drawing on our understanding of the contextual barriers present across primary and specialist healthcare contexts, implementers' own account of programme mechanisms, and Normalisation Process Theory, we formulated a second revised programme theory:

Participatory self‐assessment approaches (I) enable healthcare service providers to view their practice through a ‘trauma lens’ and understand where ‐ and why ‐ changes are needed (M1). The approach's non‐judgemental, immersive nature helps staff engage in the sense‐making work necessary for change to become successfully normalised or embedded in routine practice, increasing its coherence and sustainability ^24^ (M2). In a supportive organisational context with dedicated time and resources for training and reflective practice (C), this promotes lasting changes to practice, achieving better outcomes for patients (O).

### 4.3 Scope and limitations

This realist review was designed to investigate the role of child sexual abuse/exploitation self‐assessment tools in improving health care professionals' knowledge and confidence of child sexual abuse/exploitation, asking ‘Which self‐assessment tools and protocols are currently in use?’, ‘Does the available literature on these tools/protocols yield transferable theories regarding how, for whom and under what circumstances such tools/protocols are effective?’, and exploring what ‘good practice’ in the use of such tools entails.

While extensive efforts were made to identify all potentially relevant literature, researchers encountered a dearth of articles specifically in relation to child sexual abuse/exploitation. In addition to this, the majority of items which were judged to be relevant for inclusion lacked the kind of rich, detailed information about implementation contexts that would allow for a more robust mapping of CMOcs – identified programme theories may, therefore, represent a more ‘idealised’ and provisional account in need of further interrogation and refinement.

However, this dearth of evidence represents a significant finding or ‘answer’ of sorts, underlining a critical lacuna in the literature and a potential gap in medical curricula/CPD. It is important to note that researchers' initial research questions emerged in consultation with practitioners/advocates for child sexual abuse/exploitation survivors and from prior research articulating adult survivors' unmet needs in relation to healthcare [6, 7]. While it is not a foregone conclusion that dedicated self‐assessment tools or protocols are needed as part of an adequate healthcare response to adult‐child sexual abuse/exploitation survivors (as opposed to employing trauma‐informed practice/care or other umbrella approaches), it is a question that merits further exploration, ideally as part of a participatory research programme with health care professionals, advocates and survivors.

One methodologically salient barrier that emerged during the full‐text review and data extraction was a lack of ‘thick’ descriptive information about programme processes and implementation contexts in the reviewed studies and articles. Not all outputs that proceeded to this review stage yielded sufficiently rich, detailed information to derive insights about CMO configurations. To draw inferences about underlying causal mechanisms and arrive at a deeper explanatory analysis – rather than merely reporting on the notional or idealised programme theories advanced by programme implementers – we, therefore, employed supplementary realist tools such as interpretative abduction [29]. Abduction, in a realist sense, means “inference to the best explanation”, involving an “iterative process of examining evidence and developing hunches or ideas about the causal factors linked to that evidence” ([29]: 6).

Available evidence suggests that PREMIS was commonly used to document knowledge and confidence deficits, track progress over time and demonstrate differences between groups. Notably, there was limited evidence regarding the use of PREMIS for one of its specified purposes: supporting self‐directed learning by sensitising healthcare professionals to the issue and alerting them to gaps in their present knowledge. Instead, researchers discerned an implicit, intervening mechanism mediating changes: promoting accountability by highlighting areas where the current curriculum/training strategy is lacking.

Conversely, our revised programme theory for the organisational self‐assessments reviewed suggests that they may fulfil a sensitising and sense‐making role for staff, allowing practice changes to normalise.

While it is plausible that the identified mechanisms are transferable, none of the tools identified via the review were developed specifically in relation to child sexual abuse/exploitation. Given the reported difficulties in encouraging enquiry about domestic abuse, in part due to its sensitive nature as a subject, it is likely that developing an analogous tool for assessing knowledge, attitudes, beliefs, and behaviours could present additional challenges.

Equally, it is important to note that PREMIS was originally developed and tested to assess physicians' competencies in relation to domestic abuse, and the developers noted that further research would be needed to evaluate the tools' psychometric properties in nurse and other health care professionals [26].

Interestingly, we found limited evidence of self‐assessments as an intervention per se; in each of the eight articles/studies included in our final synthesis, these were implemented as part of wider educational efforts or to highlight educational/practice deficiencies. This finding may reflect the wider literature on self‐assessment, which suggests that self‐assessments are more accurate when they incorporate detailed guidance and external benchmarks [20]. Accordingly, self‐assessment may be more likely to yield positive behavioural/practice outcomes when scaffolded by complementary educational strategies such as didactic or skills‐based training.

Contextual barriers identified during full‐text review and data extraction included constrained time and resources for training (Brady, 2018), competing policy initiatives [30] and staff burnout [27]. To a certain extent, sustainability is contingent on “interventions being congruent with existing policy‐driven demands placed on clinicians” ([28],[30]: 91). Similarly, as research with clinicians has demonstrated that they “infrequently enquire about domestic abuse, typically citing discomfort in raising the issue” ([30],[31]: 2), interventions designed to promote ‘trauma work’, even if only in the form of facilitating disclosures and making referrals, will need to anticipate and negotiate this resistance – for example by incorporating time and resources for debriefing and reflective practice.

### 4.4 Implications and conclusion

Our review identified a striking dearth of child sexual abuse and exploitation‐related self‐assessment tools, and a corresponding lack of inquiry as to why this is such an under‐developed and under‐researched area, particularly given the relative availability of domestic abuse and generically trauma‐informed tools and protocols. Our findings suggest that more research is needed to understand how self‐assessment tools could potentially play a role in increasing nurses and other healthcare professionals' knowledge and confidence in relation to child sexual abuse/exploitation and that further research is warranted.

Available evidence indicates that ‘good practice’ in the use of trauma and abuse‐related self‐assessment tools/protocols necessitates strong leadership and adept management, including external guidance and benchmarking, complementary educational strategies and an organisational context whose leadership is responsive to feedback and willing to invest the time and resources necessary to surmount contextual barriers such as high staff turnover and burnout.

Given ongoing debates about the merits of ‘umbrella’ approaches to trauma and adversity, and the unique stigma surrounding child sexual abuse, this points to fruitful areas for future research.

## AUTHOR CONTRIBUTIONS

**Olumide Adisa**: Conceptualisation, supervision, funding acquisition, visualisation, writing–original draft, writing–review & editing. **Katie Tyrrell**: Writing—original draft; writing—review and editing. **Katherine Allen**: Visualization; writing—original draft; writing—review and editing.

## CONFLICT OF INTEREST STATEMENT

The authors declare no conflicts of interest.

## Data Availability

The data that support the findings of this study are available from the corresponding author upon reasonable request.

## Footnotes

* Covidence is a web‐based systematic review tool designed to facilitate abstract screening, full‐text review, data extraction, and analysis. It is widely used and accepted; it is the primary tool used by Cochrane reviewers and is recommended by Cochrane.
